# Indents

**Published:** 1914-10-15

**Authors:** 


### INDENTS.

☞Brief Articles of Technical or Scientific Value will receive notice and comment. Contributions invited.

*The Short Road to Success.* We have, at various times, commented on the foreign opinion of American physicians and the brazen effrontry with which it is attempted to foist proprietary preparations on American physicians. More recently space has been given to a discussion of the so-called "painless childbirth" method of Freiburg, now being exploited by an American magazine. In view of this situation, the following comment by an American medical journal (the *Cleveland Medical Journal*) seems exceedingly appropriate:

"With the recent acknowledgment from *McClure's Magazine* (The Journal A. M. A., June 13, 1914, p. 1912) that their article "Painless Childbirth", was technically correct and read by Prof. Kroenig and Doctor Gauss, we feel tempted, along with others, to lament the changes coming over many of the German medical men of today. The old German ideals of deep study, of verity and of thoroughness —what, may we ask, is becoming of them? Much of the German medical literature of today is flooded with superficial studies and absurd deductions on this, that and the other subject intended to attract the public eye. At Berlin and Vienna are arranged elaborate postgraduate courses of from one to four weeks or longer, principally shorter. They are ground out to your order, in English or German, at so many "simoleons" per minute and at the end, a much embossed much begilded diploma will be duly handed out to the participant. We are glad to say that this does not apply to the work of many of the worthy professors in these great founts of medical knowledge, but sad to relate it is only too true of many of the courses offered. And what is even worse, Americans are the particular game and laughing stock of all too many of those private-dozents, ausserordentlich professors, geheimrats, et cetera. American medicine has not yet recovered from the shock of Friedman's Golden

Fraud and we are pained to learn that Profeessor Kroenig would also add his name to those men desiring dollars rather than wisdom and the honor of their fellow workers. We say this in spite of the recent denial from him as given in the *Lancet Clinic.* The German medical man has but to put a few titles before his name, appear in America and he feels himself called upon to burst out in public print to his heart's desire, and his pockets overflowing—witness the example of a noted orthopedist a few years ago and just lately of an aurist. It is said that our remarkable antisyphilitic remedy, salvarsan, costs nine marks (about two dollars) a kilogram to manufacture and is sold to the public at the *moderate* price of nine thousane marks (about two thousand dollars) per kilogram. True, it is also said, that all the profits are turned over to the institute at Frankfort; but what, might we ask, would happen if the Rockefeller or Pasteur Institutes should adopt such tactics with so valuable a remedy? Think of the hundreds and hundreds of dispensary patients denied this drug because of its prohibitive cost. Is it possible that the German medical man of today is, in his desire for material gain, forgetting the higher ideals of research, of teaching others and of healing the sick? There is much food for thought for his American colleagues and we deeply hope that medical Germany will take no chance to besmirch that high reputation of which she can be so justly proud."—*Journal A. M. A.*, Aug. 15, 1914.

*Anesthesia of the Dentin with Albargin.* After an instructive review of the many methods which have been suggested and abandoned, and those still in vogue for the desensitization of hypersensitive dentin, the writer enumerates the following conditions for a satisfactory dentinal anesthetic: It must not injure the pulp or the solid constituents of the tooth; it must not discolor the tooth; be applicable with ease, and absolutely efficient; it must not injure the soft tissues if brought into contact therewith; its action must be permanent. Moreover, it is desirable that its application require a short time, call for no special precautionary measures, or special instruments, and that its price be low. While fulfilling some of these conditions, silver nitrate is not fully satisfactory. All the conditions enumerated, however, are fulfilled by the silver preparation albargin, which is a combination of silver nitrate with gelatose, and is conveniently applied with a double-headed ball and spatula burnisher. It can be satisfactorily applied in hyperesthesia of the dentin at the cervical margin with incipient caries, in erosions, in hypersensitive dentin which is to be excavated in cavity preparation, in grinding teeth for crown and bridge work, in hypersensitive dentin which for some reason or other is not to be excavated, in hypersensitivity due to close bite or attrition, in carious deciduous teeth in children, in aphthae and all inflammatory conditions in which silver nitrate is indicated. A fragment of an albargin tablet is pressed upon the hypersensitive field of operation, where it is left adhering for the desired length of time. To prevent its dissolution, the field is kept dry. If desirable, a solution of any suitable strength can be made with distilled water. It is rarely necessary, according to the writer, to prolong the application beyond a period of five minutes, or to repeat the application. He also observed that after a single application of albargin, caries in deciduous teeth had not progressed further in two years, and that hyperesthesia of the dentin at the cervical margin had not returned after the same period of time. In other cases, however, the effect lasted only for one year. Hardly any discoloration was noticeable. In excavating hypersensitive cavities or in grinding down teeth, the effect of albargin is always more prolonged than that of phenol, cocain, perhydrol, or any other medicaments.

In summing up, Lichtwitz unconditionally advocates the use of albargin instead of silver nitrate, emphasizing its effectiveness, convenient and harmless applicability, and absence of discoloration.—Dr. Lichtwitz, *Cosmos* for August.

*Tooth Detergents.* *To the Editor:*—Has THE JOURNAL published any report on the detergent and antiseptic effects of the various preparations advertised for cleansing the teeth? If not, would it not be well in the interest of many readers who must wish accurate information on the selection of an effective agent to have the Council examine and report on this question? S. T. A.

ANSWER.—THE JOURNAL has published recently in the Department of Therapeutics (Nov. 8 and 15, 1913, pp. 1719 and 1812) an article on oral cleanliness which discusses the principles involved in the use of tooth-powders and other preparations for cleansing the teeth. One of the constituents of many tooth-powders is soap, and this ought to be a harmless substance except for the objection to its alkalinity. The reason for employing an alkaline substance seems to be that it is recognized that dental decay is due to the effect of acids formed by fermentation. These acids, however, do not accumulate on the teeth in the free state, but unite with the lime-salts of the enamel or dentine as soon as they are formed. The alkalinity of the ordinary tooth-powder can be of little service because its occasional application can do nothing to check the action of acids which are continuously formed during the intervals. Moreover, the use of an alkaline application to the teeth may be a positive detriment by depressing the secretion and alkalinity of the saliva on the principle that a secretion which is no longer needed ceases to be formed. Pickerill ("Stomatology in General Practice," p. 100) has shown that alkalies applied to the teeth and gums reduce both the amount and alkalinity of the saliva secreted subsequently to the application of the alkali, while the reverse effect is produced by acids. Hence he recommends a weakly acid application rather than the alkali. Another reason for the use of acid has been advanced by W. J. Gies (*Household Art Review*, May 1913, p. 12; THE JOURNAL, Nov. 8, 1913, p. 1719), who found that alkaline applications did not readily dissolve the mucus which retained the fermenting food on the teeth and hence were not efficient cleansers. He also advocates the substitution of acid solutions. Pickerill has found that daily rubbing the teeth with a weak solution of tartaric acid did not remove any appreciable amount of the material of the tooth. It seems therefore, that tooth detergents should be made of weak acids rather than weak alkalies.

An antiseptic is usually added to these preparations with a view to hindering the proliferation of the numerous microorganisms which are found in the mouth or introduced with the food. It is time that physicians should have clear ideas as to what can be expected from antiseptics applied to the oral cavity. To disinfect the mouth would be a difficult matter, and in practice is not worth attempting, unless by the direct work of a skillful operator. To give the patient an antiseptic with which to treat the mouth is worse than useless. If the object is to prevent putrefaction or fermentation it is much simpler to remove mechanically the fermentable material than to attempt to preserve it with an antiseptic. Pickerill suggests that not only is an antiseptic which is in contact with the organisms not more than three minutes a day of no use, but not improbably it has the reverse effect —that of producing a more vigorous and resisting strain of organisms due to the *slight* and *intermittent* opposition to their growth and to the survival of the fittest. This author states that for a chronic and slowly progressing lesion like caries of the teeth, the intermittent use of antiseptics is of no value. Their use should be limited to acute conditions and then they should be used frequently and in full strength that is, as strong as can be tolerated, with the object of rapidly reducing the number and virulence of the organisms. —*Journal A. M. A.*, July 4, 1914.

*The War and the Drug-Supply.* While American life has not been at once affected in any wide-spread dramatic way by the European catastrophe, all sorts and conditions of men are likely to find it touching them at unexpected points. Physicians, for instance, may find that they are hampered in the writing of prescriptions by scarcity of some drug. This country doubtless would suffer little inconvenience beyond the increase in price if compelled to rely on the resources of this continent so far as most inorganic chemicals and biologic preparations are concerned. As for vegetable drugs, however, aside from cascara, hydrastis, and podophyllum, few of therapeutic importance are produced in the United States. This country depends largely on European manufacturers for finished preparations and on European middlemen for crude drugs. Crude drugs from the Far East, such as buchu, cinchona, senna, asafetida, rhubarb, opium and aloes, come to us largely through the London and Amsterdam markets. A comparatively small amount of cinchona comes from South America, and, if access to the London and Amsterdam markets is shut off the South American drug might be obtained direct. While the prices for these substances have advanced, the supply should not be altogether cut off by this war unless it should spread to the East and thus limit production. This year's opium crop in southeastern Europe is said to have been greatly damaged by the recent war in the Balkan States and Turkey. Of imported synthetic drugs and alkaloids, by far the largest proportion come from Germany. Ophthalmologists are likely to feel the result of the interruption of trade with Germany in the withdrawal of supplies of physostigmin, pilocarpin, atropin and homatropin. Other important drugs of German manufacture are strychnin, quinin, caffein, cocain, theobromin, formaldehyd and salvarsan. The involvement of France, the chief source of supply of tartaric acid, and of Austria-Hungary, which with France and the Balkan States supplies us with the essential oils, will affect the drug supply much less than the isolation of Germany.

We may reckon some gain along with the loss if the war leads to a scrutiny with a view to revision of the United States patent laws. which at present favor the importer at the expense of the American public. The patent laws of

Great Britain and some other countries provide that the holder of a patent must, under penalty of forfeiture, begin manufacture of the patented product within the country in a reasonable period of time from the issuance of the patent. An American branch of at least one German house had begun the manufacture of a synthetic product before the war. Others may find it worth while to manufacture on American soil if the war continues; but if our patent laws had contained a provision similar to that of the British law, we would not now be threatened with temporary shortage or stoppage of therapeutic supplies. Our laws, in addition, permit the patenting, not merely of the process, but also of the product. Therefore the American market is altogether dependent for any given patented product on a single foreign manufacturer. Revision of our patent laws may become a necessity if the European war is not over soon. Another result may be an investigation of the possibility of producing in this country a larger proportion of the drugs used here. The Department of Agriculture has long maintained that, so far as soil and climate are concerned, the conditions are favorable for the growing in this country of many of the drug-plants now imported from abroad. In view of the uncertainty whether or not, under practical conditions, the yield could be marketed profitably, cultivators have naturally been somewhat reluctant to experiment with untried crops; but the stimulus of war prices doubtless will induce some growers to make a trial that will demonstrate which drug-plants are commercially profitable.—*Journal A. M. A.*, August 15, 1914.

*Importance of a Complete Diagnosis.* The human masticatory apparatus is nothing but a machine intended for the trituration of food for its proper introduction into the alimentary canal. We would not think of going to a tailor and asking him to fit us a sleeve of a coat. He might make ever so perfect a sleeve and we would not have the body of the coat complete. We would only have a perfect sleeve. When the patient comes and says, I want you to fix this tooth, he is asking for the sleeve of the coat. You have to look at the whole masticatory apparatus. What is the order? Make a diagnosis, as has been said. Take everything into consideration and then plan your work accordingly. Make a good model. Plan the work of taking impressions and securing casts and study those, not only in the privacy of your laboratory after the patient has gone, but by taking these casts into the operating room when the patient returns and by comparing, looking at the casts and making comparisons with the mouth after you have had time to study the case over. You can figure out what kind of apparatus or what kind of restoration will be necessary and will give that patient the best service. You can find out far better that way than if you attempted to do it by an examination at the first sitting. Many men I know in practice will receive a patient in their office, have him seated in the chair, look over his mouth carefully and say, now we will do this and this and this, and what they outline there they carry out to the letter; but if they had made their models and had time to sit down and study the case, consider the forces that would be exerted upon the fillings, inlays, crowns, bridges or whatever appliances were being placed in the mouth, they might change their minds. An entirely different class of restoration might be developed to the better advantage of the patient than if they had attempted to do it in a haphazard sort of way or do it quickly. You will come to this point some day that you will not place an inlay in a tooth or construct a crown or bridge without considering the anatomical relation of that tooth to the opposing teeth, constructing a plane that will help to hold that tooth in its proper position and help to maintain contact of that tooth with those in opposition. When we consider, as I firmly believe, that 95 percent. of all cases of pyorrhea originate in loss of contact of the teeth, you can see how necessary it is to associate this plane, so that the teeth would be jammed together from the third molar on one side to the third molar on the other, rather than have the teeth so that they will separate under masticatory stress.—Dr. Prothro, in *Dominion Journal*.

*Pulp Devitalization Condemned.* I strongly assert that there are too many pulps devitalized. I have heard it said a pulp will die under a metal cap crown cemented on a tooth. I have in times past trimmed away the entire enamel from a molar and placed upon that for the support of a bridge a metal cap, and I won't say that none of them have died because I could not prove it, but I do not believe that they have died. We do know that thousands of teeth have suffered through the insertion of cement over sensitive or over a thin layer of dentin in the insertion of fillings. That is a little different matter from the tearing away of not only the enamel but a portion of the dentin from a molar tooth or any tooth upon which is to be placed the cap crown, because when the cement is placed there you are driving into the pulp something that will possibly cause its death.—Dr. Magee, in *Dominion Journal*.

*Value of Mask Over Surgeon's Mouth.* In order to demonstrate the value of a mask over the surgeon's mouth during operations, Candler carried out the following experiments with bacillus prodigiosus: With ordinary breathing and quiet speaking, no bacilli left the mouth. Coughing for two minutes caused only a few bacilli to be emitted, and these were controlled by a mask of eight layers of gauze, one of four layers being insufficient. Sneezing was shown to be a most dangerous source of infection from the mouth, one sneeze, and that an artificial one, producing 130 colonies of micro-organisms in a circle of $3\frac{1}{2}$ inches diameter at a distance of 18 inches. A mask of eight layers is not sufficient to keep back all organisms during prolonged sneezing, although it reduces the infection considerably.

There was, however, no growth on a plate exposed to a sneeze through such a mask.—*Brit. Med. Journal.* per *Journ. Amer. Med. Association.*

*Securing Perfect Wax Models for Cast Gold Inlays.* In order to secure a perfect wax model for a cast gold inlay, the wax is first trimmed to the desired shape with carving and modeling instruments. All accessible surfaces of the wax model are then wiped with a pellet of cotton dipped in oil of cajuput. The approximal surfaces are smoothed to below the gingival margin with a fine strip dipped in this oil.—*Oesterreich. Zeitschr. f. Stomatologie.*

*Polishing Vulcanite Dentures.* After having filed and scraped a vulcanite plate, instead of using any sandpaper, a mixture of one part of emery powder and three parts of powdered pumice is employed for finishing and polishing, affording considerable economy in time.—*Le Laboratoire et le Progres Dentaire.*

*To Remove Teeth From a Rubber Plate Without Danger of Cracking or Etching the Teeth.* Boil the plate in glycerine, in a porcelain pan till it smokes; the teeth will then come away clean and free from discoloration. Put them back in the glycerin till cold to anneal them, and when cool wash in warm water. They will be as bright as when new, and also free from grease. When cold the glycerine can be bottled for further use. Unlike oil, it is odorless when used hot.—*Ohio Dental Journal.*

*Clean Impression Trays.* Clean trays and dip them into a pan of hot wax, then hang them up to dry. This will give the trays a thin coating of wax. Take impression in the usual manner and remove plaster from the tray by heating it over flame. Dip tray in wax solution again and hang it in place for future use. This will insure bright, clean looking impression trays.—E. Eustice, in *Review*.

*Model Cement.* I have had the experience of having my models dropped and broken. You have experienced the same thing probably. It is a very difficult matter to unite the parts in such a way that they will not be unsightly. There is nothing new about this celluloid solution, as it is nothing more than plain celluloid dissolved in ether. The other bottle contains celluloid dissolved in spirit of camphor and alcohol. If the parts are entirely dry and free from moisture, simply place a little drop of the solution, press the parts together and let them dry. In less than a day the liquids will have evaporated, leaving the celluloid and making a clean joint. You can scarcely see where the break occurred.—Dr. Guilford, in *Garretsonian*.

